# Anaplastic Multiple Myeloma: Case Series and Literature Review

**DOI:** 10.36502/2022/asjbccr.6255

**Published:** 2022-01-15

**Authors:** Jian Wu, Emily Chu, Cristiana Costa Chase, Taewoong Choi, Cristina Gasparetto, Ken Young, Yubin Kang

**Affiliations:** 1 Division of Hematologic Malignancies and Cellular Therapy, Department of Medicine, Duke University Medical Center, Durham, North Carolina, USA; 2 Division of Hematopathology, Department of Pathology, Duke University Medical Center, Durham, North Carolina, USA

**Keywords:** Anaplastic Multiple Myeloma, Multiple Myeloma, Case Series, Treatment, Prognosis

## Abstract

**Background:**

Anaplastic multiple myeloma (AMM) is a very rare but distinct subtype of multiple myeloma (MM) with an extremely poor prognosis. Due to its rarity, AMM lacks detailed descriptions and clear definitions. Moreover, there is no consensus on the treatment and evidence suggests that AMM responds poorly to several novel therapies. We conducted a literature review and retrospective case series to determine clinical characteristics, pathological features, and outcomes of AMM.

**Case Presentation:**

Published case reports and case series of AMM since 1983 were systematically extracted and reviewed. A total of 52 patients with AMM were reported in the PUBMED since 1983, including 26 males (50%) and 26 females (50%). The age ranged from 29 years old to 85 years old, with a mean age of 57.02 years old. Most of the patients presented with bone pain (23, 44.2%), fatigue (18, 34.6%), plasmacytoma (18, 34.6%) and weight loss (7, 13.5%). The median survival of the patients was 4 months. To investigate the outcomes of patients with AMM in the current era of treatment, a series of 14 patients with AMM diagnosed at our institute between December 2012 and July 2021was retrospectively analyzed. Our retrospective case series consisted of 12 males (85.7%) and 2 females (14.3%), with a mean age of 59 years old. Most of our AMM patients displayed bone lytic lesions as a common manifestation. The common cytogenetic abnormality was 1q amplification. All patients received standard combination chemotherapy consisting of proteasome inhibitors and/or immunomodulatory agents, and half of the patients underwent autologous hematopoietic stem cell transplantation. The median progression-free survival (PFS) and overall survival (OS) for our 14 AMM patients were 0.84 years and 1.52 years, respectively, which was significantly worse than the regular MM patients treated at our institute from 2003–2013 who had a PFS of 2.28 years and OS of 4.92 years.

**Conclusions:**

AMM is a very rare, morphologically distinct variant of MM. It has adverse cytogenetics and an aggressive course. It is often resistant to standard chemotherapy and presents with an extremely low survival rate.

## Background

Anaplastic multiple myeloma (AMM), also known as plasmablastic plasma cell myeloma, is an extremely rare disease with an aggressive clinical course and unfavorable prognosis. It was first described in 1983 by Foucar et al [1], who reported two patients who developed anaplastic myeloma associated with the extramedullary disease with a prominent intra-abdominal and retroperitoneal tumor mass. AMM can present de novo at disease onset, but can also transform from conventional plasma cell myeloma, and the transformation typically occurs between the first and fourth year of the diagnosis of multiple myeloma (MM). The prognosis of patients with AMM is extremely poor with current treatment and most of AMM patients live for only a few months after diagnosis [2]. The exact incidence of AMM is not clear. It was estimated that in approximately 2.6% of plasma cell myelomas, the morphology of the plasma cells was highly pleomorphic, quite anaplastic, and may resemble that of metastatic tumor cells. Due to the rarity of the disease, only a limited number of AMM cases have been reported and there is a lack of a systemic approach for the understanding and the management of this disease. In the current study, we conducted a literature review and performed a retrospective study of 14 AMM patients seen at our institute in an effort to further analyze the clinical and pathological features of AMM patients and to provide some suggestions for diagnosis and treatment.

### Cases Presentation

#### Literature Review:

We searched PubMed for articles with the keywords: anaplastic myeloma and plasmablastic multiple myeloma; we then filtered for articles published in the English language and involved human patients since 1983. We included all original case reports and case series, and all articles were individually reviewed. Clinical characteristics and outcomes were extracted.

We searched PUBMED for publications reporting AMM case(s) between 1983 and 2021. We found twenty-three articles that met the criteria [1–23]. A total of 52 anaplastic myeloma cases were reported during the period between 1983 and 2021: 26 were male and 26 were female with the mean age being years old (Table-1). AMM occurred in all ethnic groups. Unlike plasmablastic lymphoma, most AMM patients did not have pre-existing HIV conditions. The initial presentation of AMM in most patients consisted of fatigue (34.6%), bone pain (44.2%), and weight loss (13.5%). It is also interesting to note that thrombocytopenia was in the initial presentation in 9.6% of patients. Eighteen cases (34.6%) presented with extramedullary plasmacytoma involving right brachial plexus [4], pancreatic involvement [5], brain [8], soft tissue [18], intraperitoneal mass [11], liver [19], and left parotid region [23], etc. IgA was the most common M protein subtype (32.7%) and 1q amplification was the most prevalent cytogenic abnormality (23%). Patients with AMM were typically treated with standard regular MM regimens such as VCD (Bortezomib + Cyclophosphamide+ Dexamethasone), or VAD (Vincristine + Adriamycin+ Dexamethasone), and /or VTD (Bortezomib + Thalidomide + Dexamethasone), and in 17.3% of patients followed by consolidation with autologous hematopoietic stem cell transplant (HSCT). Unfortunately, ~44% of patients with AMM did not survive the past 6 months and only 7.6% of patients survived over 24 months. The median survival was 4 months.

#### Cases Series:

To investigate the outcomes of patients with AMM in the current era of treatment, we performed a retrospective case study at our institute. The retrospective chart review was performed in accordance with the ethical standards of Duke University Institutional Review Board (IRB) Committees on human experimentation and was approved by the Duke IRB committee. We queried and searched Duke electronic medical records from December 2012 to July 2021 and identified 14 patients with AMM to be included for the study. The patient inclusion criteria are having a diagnosis of AMM confirmed at Duke; and having medical records available that include laboratory data at the time of diagnosis, treatment regiment, and survival status. Detailed information regarding clinical presentation, laboratory finding, imaging tests, and treatment were extracted and included: age at the time of diagnosis, gender, race, date of diagnosis, laboratory values, PET CT and other imaging, bone marrow biopsy (plasma cell percentage), disease stage (International Stage Index), cytogenetics (karyotype and FISH), treatment regimens including hematopoietic stem cell transplant, progression-free survival, and overall survival.

**Fig-1:**
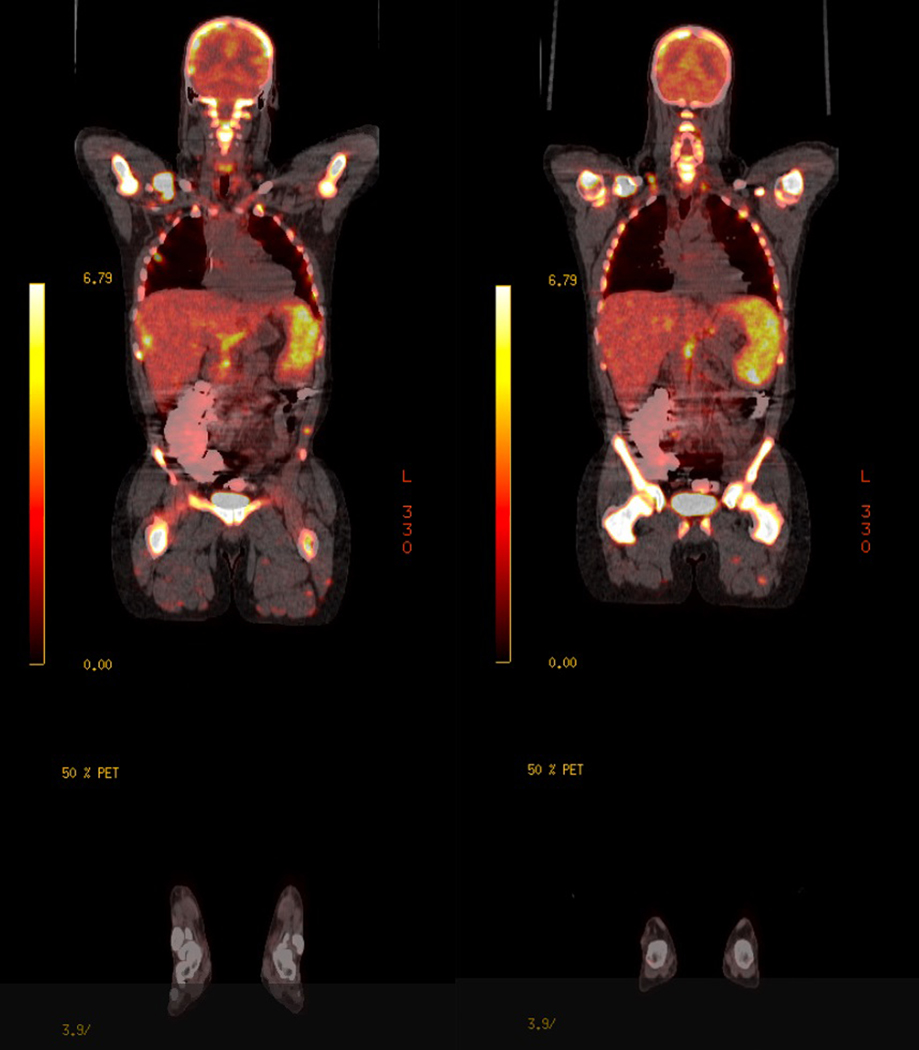
18F-FDG PET/CT in one patient with multiple myeloma 18F-FDG PET/CT scan demonstrates intense hypermetabolic FDG activity in patient with anaplastic multiple myeloma. 18F-FDG, 18F-flurodeoxyglucose, PET, positron emission tomography; CT, computed tomography.

**Fig-2:**
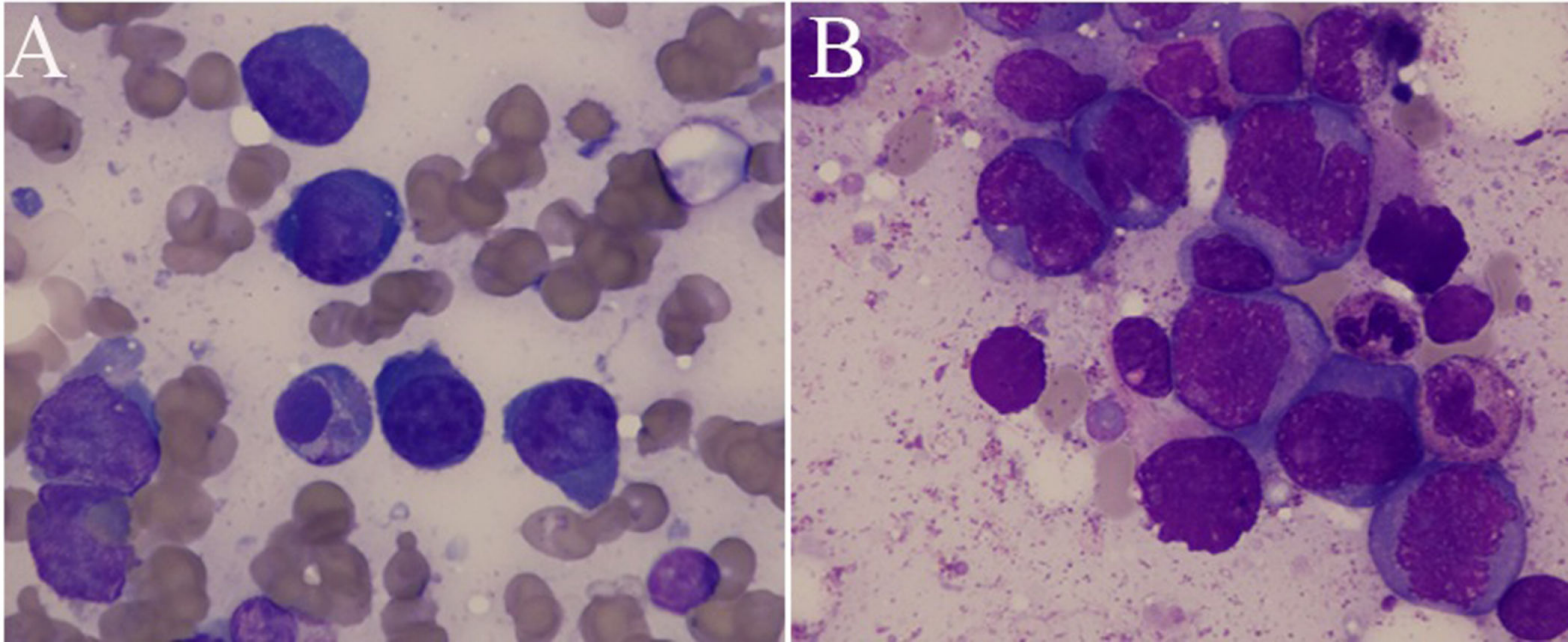
Peripheral blood and bone marrow evaluation showed sheets of neoplastic cells with many anaplastic forms (A) Peripheral blood smear (50×) showing morphological changes in anaplastic myeloma cells. (B) On bone marrow aspirate, the anaplastic cells resembled dysplastic megakaryocytes with purple-bluish granular cytoplasm and bizarre or separated nuclei.

**Fig-3:**
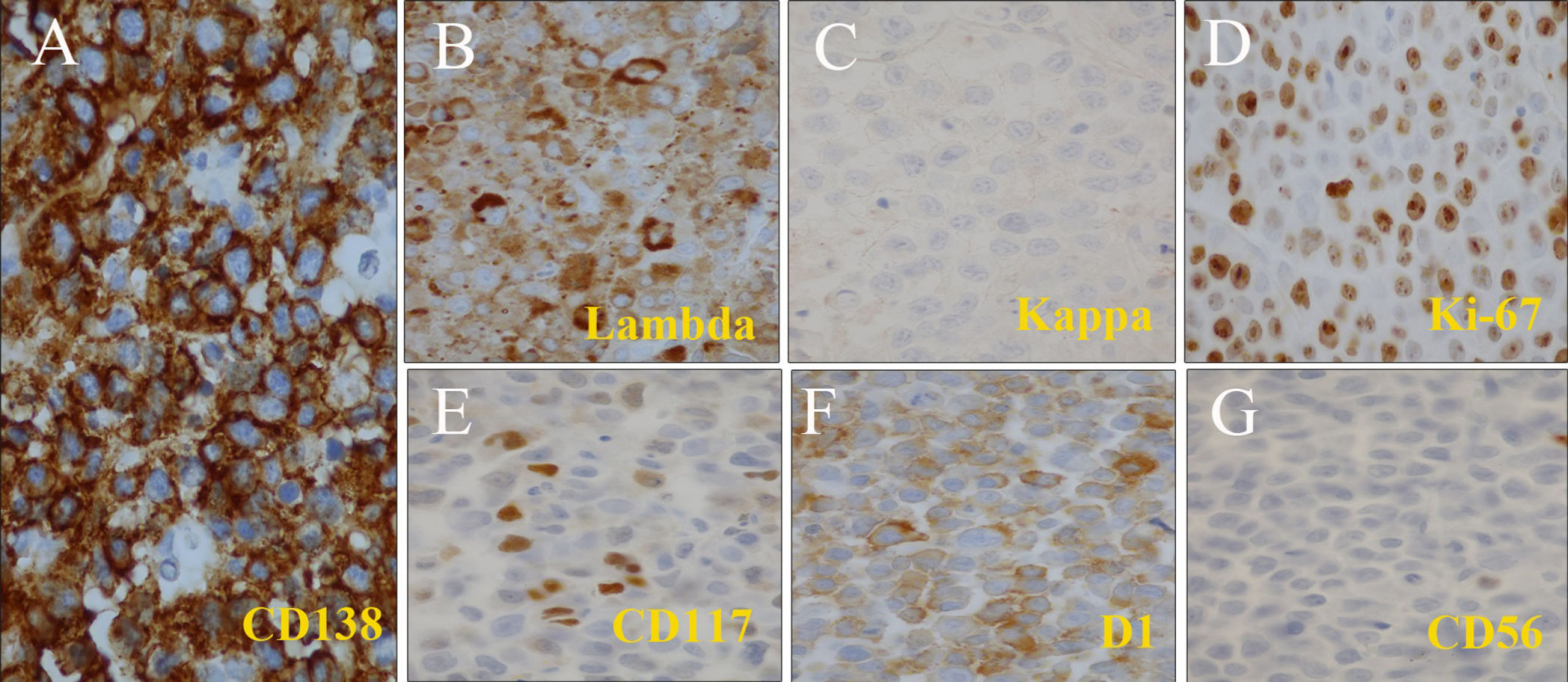
Pathological findings of bone marrow at diagnosis The neoplastic cells were positive for CD138 (A) and cytoplasmic lambda light chain (B), and negative for cytoplasmic kappa light chain (C). The neoplastic cells were positive for Ki67 (D), CD117 (E) but negative for CD56 (F).

We identified 14 patients with AMM at our institute between December 2012 to July 2021. Male patients accounted for 85.7% of the cases with only 2 female patients in our cohort. The mean age at diagnosis was 59 years old, ranging from 46 to 85 years old. All ethnic groups (Caucasian, African American, Hispanic, and Asian) are affected. 9 patients (64.3%) presented with de novo AMM, whereas in 5 patients (35.7%) AMM was transformed from pre-existing myeloma and presented at the time of myeloma relapse. The majority of the AMM patients (12 patients, 85.7%) demonstrated extensive osseous lytic lesions and/or plasmacytoma. The diffuse fluorodeoxyglucose avidity and several bones and extramedullary lesions in one of the AMM patients were shown in the PET CT scan (Fig-1). IgA subtype accounted for 28.6% of the patients. Amplification of 1q occurred in 50% of the patients and is the most common genetic abnormality in AMM (Table-2). The tumor cells were primitive-appearing and anaplastic and could resemble poorly differentiated immunoblast-like cells (Fig-2). The infiltrating plasma cells were positive for CD138 with light chain restriction. The nuclear Ki-67 expression could be high, in some cases, the nuclear Ki-67 expression could reach over 90%, indicating a high proliferation fraction. The expression of CD117, cyclin D1, and CD56 could be highly variable (Fig-3) and the plasma cells could also be positive for CD20 (data not shown).

AMM patients were treated with standard MM regimen including VRD (Bortezomib + Revlimid + Dexamethasone), or VCD (Bortezomib + Cyclophosphamide + Dexamethasone), and /or VD-PACE regimen. 7 patients (50%) received high dose melphalan conditioning followed by autologous HSCT. Despite receiving standard MM treatment, the median progression-free survival (PFS) for our 14 AMM patients was 0.84 years, and the median overall survival (OS) was 1.52 years.

**Fig-4:**
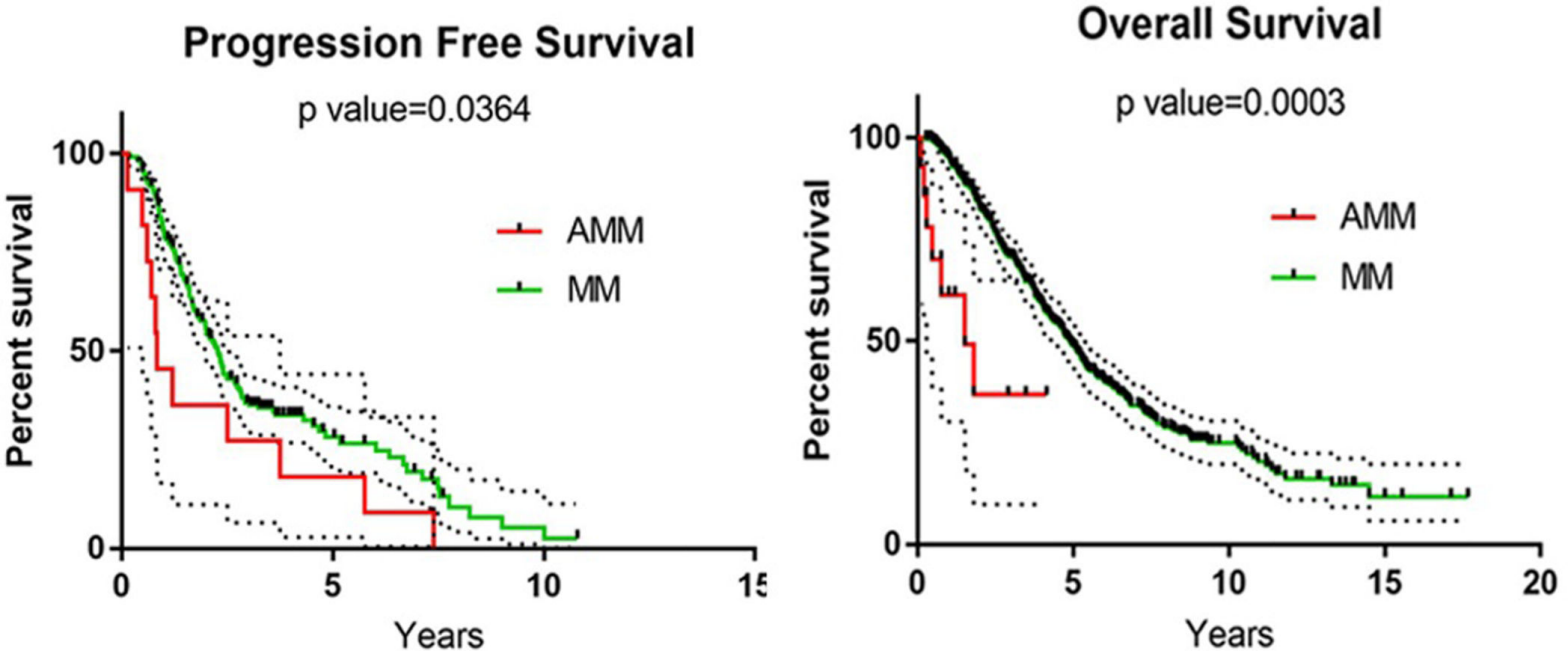
The progression free survival and overall survival analysis between AMM and regular MM

We compared the treatment outcomes of our 14 AMM patients with a historical dataset of 393 regular MM patients treated at our institute from 2003 to 2013. The patient characteristics and treatment of our 393 regular MM patients were summarized in Table-3. Although our regular MM patients were treated 10 years earlier, the PFS and OS of the patients with AMM were significantly worse than those in patients with regular MM: PFS was 0.84 years and 2.28 years for AMM and regular MM, respectively (p=0.0364). The OS was 1.52years and 4.92 years for AMM and regular MM, respectively (p=0.0003) (Fig-4).

## Discussion and Conclusions

In the current study, we performed a systematic literature review as well as retrospective cohort study in an effort to define clinical characteristics, prognosis, and outcomes of patients with AMM. We found that AMM affects both males and females and all ethnic groups. AMM occurs in a much younger patient population (median age of 57.02 years old) compared to conventional multiple myeloma (median age of 69 years old) [24]. Anemia was one of the most common presentations of AMM and thrombocytopenia occurred in 9.6% of AMM patients at presentation, which likely reflects the aggressiveness of the disease and the extensive bone marrow infiltration by anaplastic plasma cells [21]. AMM can develop de novo or transform from pre-existing regular multiple myeloma. In contrast to regular multiple myeloma, IgA was the most common M protein subtype (32.7%) and 1q amplification was the most prevalent cytogenic abnormality (23%). The prognosis of AMM is dismal and very few AMM patients survived past 24 months after the diagnosis.

In our case series, osseous involvement was very common in AMM patients. It is also interesting to note that in the literature reviews, 18 of the cases presented with extramedullary plasmacytoma (34.6%) located in the right brachial plexus [4], pancreatic involvement [5], brain [8], soft tissue [18], intraperitoneal mass [11], liver [19], and left parotid region [23], etc. Extramedullary plasmacytoma (EMP) is typically seen in MM patients at disease relapse or as an aggressive disease presentation. The plasma cells in EMP have an immature and plasma-plastic appearance when compared to the plasmacytoma with bone marrow involvement. There was a research that indicates that clonal mutations of TP_53_, RB_1_, FAK, and RAS genes have been presented in 50% of patients who have EMP presentation with MM [25]. In AMM, mutation of TP53 is high, which could possibly lead to increased EMP presentation. More research must be done in order to determine the significance of EMP in AMM patients and how this should be evaluated when developing a standard course of treatment.

The cellular origin of anaplastic multiple myeloma is considered to be immature plasma cells [26]. Morphologically, the diameter of anaplastic myeloma cells is significantly enlarged by approximately twofold. The atypical pleomorphic, multinucleated morphology of anaplastic myeloma cells can mimic multinucleated carcinoma. The anaplastic cells may simulate dysplastic megakaryocytes with bluish granular, basophilic cytoplasm with bizarre multilobed nuclei; the nuclei are hyperchromatic with abnormal distribution. Anaplastic myeloma cells have moderate to abundant basophilic cytoplasm, prominent nucleoli, and intranuclear basophilic inclusions (Fig-2). Therefore, the differential diagnosis for AMM includes metastatic carcinoma, acute leukemia/myeloid dysplastic syndrome with dysplastic megakaryocytes, and plasmablastic lymphoma (PBL). The distinction between AMM and PBL can be difficult. PBL is also a rare subtype of B-lymphoid malignancy, which has pathological features that can overlap with aggressive, mature B-cell lymphomas and plasma cell neoplasms [27]. There are a number of clinicopathological features that will support a diagnosis of AMM, such as renal dysfunction, significant paraprotein level, osteolytic lesions, hypercalcemia, and diffuse bone marrow involvement [28]. The current differential diagnosis between PBL and AMM is focused on the difference in clinical manifestations, such as M-protein levels, HIV infections, and osteolytic changes [29]. Additionally, with the widespread application of FISH and next-generation sequencing, the chromosomal and genetic abnormalities that is unique to AMM such as 1q amplification and translocation of t (11;14) have made it easier to distinguish PBL from AMM.

Currently, the genetic and molecular mechanisms driving the pathogenesis and the aggressiveness of AMM remain largely uncharacterized. Due to the rarity of the disease, no studies have ever been reported determining the differences in genetic/molecular pathways and/or immune signatures between AMM and regular MM. As shown in our current study, the common chromosomal and FISH abnormalities in AMM included 1q21 amplification, 17p(p53) deletion, t (4:14), and/or chromosome 13 anomalies. 1q amplification was observed in 50% of our case series. Cyclin kinase subunit 1B (CKS1B) gene is located at chromosomal locus 1q21 and was associated with aggressive disease progression and poor prognosis in MM, even after HSCT [30]. CKS1B expression was low to undetectable in healthy subjects and in patients with monoclonal gammopathy of undetermined significance (MGUS), but significantly increased in patients with relapse/refractory MM [31]. CKS1B regulates the ubiquitination and proteasomal degradation of p27Kip1 [32,33] and plays an important role in tumor cell proliferation, survival and drug resistance [31]. AMM was associated with a significantly higher prevalence of CKS1B amplification compared with regular MM (91% vs. 34% respectively) [10]. Whether CKS1B contributes to the pathogenesis of AMM remains to be determined.

AMM is associated with an overall poor prognosis [6]. Most AMM patients have an inadequate response to conventional chemotherapy and radiotherapy [1,2,4,5,8,10,11,13,14,17,19,21,23]. In our case series, 66.7% patients experienced an unsatisfactory response to standard myeloma treatment regimens, that is VRD (Bortezomib, lenalidomide and dexamethasone) and VCD (Bortezomib, lenalidomide and dexamethasone). In two of our patients, VD-PACE regimen was used along with VRD prior to hematopoietic stem cell transplantation (HSCT) consolidation and was able to achieve remission for over 2 years. The utility of chimeric antigen receptor T-cell immunotherapy and bi-specific antibody in AMM remains to be determined.

In conclusion, our current report included 52 cases of AMM in literature review and 14 cases of AMM at our own institute, representing the largest case series of AMM ever reported. AMM is a highly malignant subtype of myeloma with a bizarre morphology and is resistant to conventional therapy. Based on our own experience, aggressive induction chemotherapy combined with novel agents followed by consolidation with autologous stem cell transplant should be offered for patients with AMM. Additional molecular and genetic pathway studies are needed to better understand the pathophysiology. Importantly, clinical trials designed specifically for AMM are urgently needed to develop more effective treatment strategies and improve the prognosis and outcomes of patients with AMM.

## Figures and Tables

**Table 1.** AMM: Patient Characteristics, Treatment, and Outcomes: literature review

	Patients (n=52) No. (%)
**Gender**	
Male	26 (50%)
Female	26 (50%)
**Age (yrs)**	
Mean	57.02
Range	29 – 85
**Race**	
Caucasian	2 (3.8%)
Asian American	18 (34.6%)
African American	2 (3.8%)
Indian	5 (9.6%)
Unknow	23 (44.2%)
**Presentation**	
Bone Pain	23(44.2%)
Fatigue	18(34.6%)
plasmacytoma	18(34.6%)
Fever	4(7.7%)
Pathologic fracture	3(5.8%)
Weight loss	7(13.5%)
Night sweats	2(3.8%)
Anemia	3(5.7%)
Mental status changed	3(5.8%)
Dyspnea	2(3.8%)
Thrombocytopenia	5(9.6%)
Renal insufficiency	1(1.9%)
Abdominal pain	3(5.7%)
**M protein**	
IgG	13 (25%)
IgA	17 (32.7%)
IgM	1 (1.9%)
IgD	1 (1.9%)
**Cytogenetics**	
TP53	9 (17.3%)
t (11;14)	7(13.4%)
t (4;14)	5(9.6%)
t (14,16)	1(1.9%)
hyperdiploidy	1(1.9%)
Del 13	8(15.4%)
1q amplification	12 (23%)
**Treatments**	
HSCT	9(17.3%)
VRD (Dexamethasone, Lenalidomide and Bortezomib)	2(3.8%)
VD (Bortezomib, Dexamethasone)	2(3.8%)
VCD (Bortezomib, Cyclophosphamide and Dexamethasone)	10(19.2)
VDD (Bortezomib, Doxorubicin, and Dexamethasone)	6(11.5%)
VTD (Bortezomib, Thalidomide and Dexamethasone)	6(11.5%)
VAD (Vincristine, Adriamycin and Dexamethasone)	1(1.9%)
BD (Bortezomib, Dexamethasone)	1(1.9%)
DVD (Daratumumab, Bortezomib and Dexamethasone)	1(1.9%)
KD (Carfilzomib, Dexamethasone)	2(3.8%)
Melphalan, cyclophosphamide, vincristine, prednisone	14(26.9%)
RCD (Lenalidomide, Cyclophosphamide and Dexamethasone)	1(1.9%)
VDD (Vincristine, Doxorubicin, and Dexamethasone)	3(5.8%)
TD (Lenalidomide, Dexamethasone)	1(1.9%)
**Outcomes**	
< 1month	2 (3.8%)
1–6 months	21(40.38%)
6–12 months	2 (3.8%)
12–24 months	12 (23.1%)
>24 months	4 (7.6%)

**Median Survival (months)**	4

**Table 2.** AMM: Patient Characteristics, Treatment, and Outcomes: Duke cases

	Patients (n=14) No. (%)
**Gender (n, %)**	
Male	12 (85.7%)
Female	2 (14.3%)
**Mean age (years)**	59
Range	46–85
**De novo vs transformation**	
De novo	9(64.3%)
Transformation	5(35.7)
**Race**	
Caucasian	6(42.9%)
African American	6(42.9%)
Hispanic	1(7.1%)
Asian	1(7.1%)
**M protein**	
IgG	7(50%)
IgA	4(28.6%)
Light chain disease	3(21.4%)
**Cytogenetics**	
1q amplification	7(50%)
TP53 deletion	3 (21.1%)
Del (13)	6 (42.9%)
t (11;14)	0
t (4;14)	3 (21.4%)
t (4;16)	1 (7.1%)
Hyperdiploidity	5 (35.7%)
**ISS stage**	
I	4(28.6%)
II	3(21.4%)
III	4(28.6%)
Unknown	3(21.4%)
**Treatment**	
VCD/VRD	12(85.7%)
VD-PACE	4(28.6%)
Autologous HSCT	7(50%)
**PFS (Median)**	0.84 years
**OS (Median)**	1.52 years

**Table 3.** Regular MM: Patient Characteristics, Treatment, and Outcomes: Duke cases

	Regular MM of Duke records (n=393)
**Gender (n, %)**	
Male	217 (55.2%)
Female	176 (44.8%)
**Age (years)**	
Median (range)	60(31–88)
**Race**	
Caucasian	246 (62.9%)
African American	129 (33%)
Asian	3 (0.8%)
Hispanic	1 (0.3%)
Native American	8 (2.0%)
Other	4 (1.0%)
**Cytogenetic stratification**	
Standard	246 (66.5%)
Intermediate	20 (5.4%)
High risk	37 (10.0%)
Unknown	67 (18.1%)
**M protein type**	
Ig G	244 (69.3%)
Ig A	84 (23.9%)
Ig M	3 (0.9%)
Ig D	2 (0.6%)
Other	21 (6.0%)
**ISS stage**	
I	74 (20.8%)
II	84 (23.7%)
III	84 (23.7%)
Unknown	113 (31.8%)
**Bone lytic lesion**	
Yes	199 (52.9%)
No	122 (32.4%)
Unknown	55 (14.6%)
**Treatment**	
HSCT	213 (63.4%)
Bortezomib	299 (98.0%)
Carfilzomib	56 (18.4%)
Ixazomib	6 (2.0%)
Thalidomide	94 (29.7%)
Lenalidomide	274 (86.7%)
Pomalidomide	62 (19.6%)
Panobinostat	7 (36.8%)
Elotuzumab	1 (5.3%)
Daratumumab	8 (42.1%)
**Treatment response**	
CR	53 (16.2%)
VGPR	93 (28.4%)
PR	122 (37.2%)
SD	22 (6.7%)
SD	22 (6.7%)
PD	21 (6.4%)
Unknown	17 (5.2%)
**PFS (Median)**	2.28 years
**OS (Median)**	4.92 years

## Abbreviations

AMM: Anaplastic Multiple Myeloma
MM: Multiple Myeloma
PFS: Progression Free Survival
OS: Overall Survival
MGUS: Monoclonal Gammopathy of Undetermined Significance
CKS1B: Cyclin Kinase Subunit 1B
EMP: Extramedullary Plasmacytoma
PBL: Plasmablastic Lymphoma
